# Phenotypic and functional heterogeneity of naïve CD8^+^ T cells in human peripheral blood during aging

**DOI:** 10.3389/fragi.2026.1765665

**Published:** 2026-02-02

**Authors:** Luca Pangrazzi, Patrizia Pehl, Lotti Hoffmann, Martin Bachmann, Gabriele Chelini, Michael Keller, Brigitte Jenewein, Maria Cavinato, Birgit Weinberger

**Affiliations:** 1 Institute for Biomedical Aging Research, University of Innsbruck, Innsbruck, Austria; 2 CNR Neuroscience Institute, Pisa, Italy

**Keywords:** adaptive immunity, aging, immunosenescence, naïve T cells, T cells

## Abstract

Naïve CD8^+^ T cells are key players of adaptive immunity, but their heterogeneity and age-related changes are not fully understood. This study aimed to compare naïve CD8^+^ T cell subsets defined by different combinations of markers, namely, N_CCR7_ (CD45RA^+^CCR7^+^), N_CD28_ (CD45RA^+^CD28^+^), N_CD27_ (CD45RA^+^CD27^+^), and phenotypically most “true-naïve”-like, N_TN_ (CD45RA^+^CCR7^+^CD28^+^CD27^+^CD57^−^). Peripheral blood was harvested from donors of various ages and the phenotype of the four subsets of naïve CD8^+^ T cells was analyzed. N_CD27_ and N_TN_ cells showed similar phenotypes with low expression of differentiation markers, pro-inflammatory cytokines, and effector molecules. Furthermore, they exhibited optimal mitochondrial fitness, low senescence markers, reduced apoptosis, and high proliferation potential. Hierarchical clustering identified cluster one including N_CD27_ and N_TN_, with lower expression of differentiation markers and pro-inflammatory molecules, and cluster 2, including N_CCR7_ and N_CD28_ cells, in which these parameters were more expressed. Age-related changes were observed in all subsets, although they were less pronounced for the N_CD27_ and N_TN_ subsets. Taken together, this study demonstrates significant heterogeneity among naïve CD8^+^ T cell subsets, with N_TN_ cells representing the most *bona fide* naïve phenotype and N_CD27_ showing a partially similar phenotype. These findings significantly enhance our understanding of naïve CD8^+^ T cell biology and function.

## Introduction

Aging is accompanied by thymic involution, a process in which the thymus undergoes progressive atrophy, structural alterations, as well as functional decline, leading to a significant decrease in the output of naïve T cells (George and Ritter, 1996; Thomas et al., 2020). As the thymus involutes, naïve T cell populations in the elderly are maintained by homeostatic proliferation of peripheral T cells (Kamimura et al., 2015). Alongside reduced naïve T cell production, increased numbers of effector/memory T cells with features of terminal differentiation are present in old age (Pangrazzi et al., 2017a; Pangrazzi et al., 2020). Persistent antigenic stimulation, including chronic viral infections, supports the accumulation of late differentiated/senescent-like T cells, particularly within the CD8 compartment (Pangrazzi and Weinberger, 2020; Appay et al., 2011; Pangrazzi et al., 2017b). The composition of the naïve CD8^+^ T cell compartment and its functionality are known to consistently change with aging, thus playing a determinant role in immunosenescence (Egorov et al., 2018). These alterations have been associated with impaired immune responses to novel antigens, therefore contributing to severity of infectious diseases in the elderly population (Seshadri et al., 2023). To achieve complete activation of naïve and memory CD8^+^ T cells, costimulatory signals are required in addition to the first signal provided by the interaction of the T cell receptor (TCR) with the MHC/peptide complex. The best-defined co-stimuli involve the interaction between the co-stimulatory receptors CD28 and CD27 expressed by T cells and their ligands CD80/CD86 and CD70 on the surface of antigen-presenting cells (APCs) (Larbi and Fulop, 2014). After persistent antigenic stimulation and several cycles of activation, expression of both CD28 and CD27 is progressively downregulated on the surface of CD8^+^ T cells (Larbi and Fulop, 2014). In addition to CD28 and CD27, naïve T cells express the lymph node homing receptor CCR7 and the CD45 isoform CD45RA (Geginat et al., 2003). Notably, Koch and colleagues defined the CD45RA^+^CCR7^+^CD27^+^CD28^+^CD57^−^ (N_TN_) subset as the most *bona fide* naïve CD8^+^ T cell population (Koch et al., 2008). Although multiple markers have been proposed to define human naïve CD8^+^ T cells, no universally accepted definition exists in the literature. This lack of consensus complicates comparisons across studies. Furthermore, no previous research has systematically evaluated and characterized the various existing definitions of naïve CD8^+^ T cells in parallel.

In this work, we assessed the expression of molecules associated with CD8^+^ T cell differentiation, cytokines, effector molecules, as well as parameters defining mitochondrial fitness, oxidative stress, and senescence, in naïve CD8^+^ T cells defined using four different combinations of markers. Furthermore, we measured DNA damage-induced apoptosis and proliferation potential of these subpopulations. Age-related changes in the phenotype of these naïve subpopulations were additionally described.

## Materials and methods

### Blood donors and isolation of PBMCs

Peripheral blood samples were obtained from systemically healthy individuals who did not suffer from diseases known to affect the immune system. Overall, our cohort included 60 donors, age range 21–84, 28 males and 32 females. The number of donors used for each individual experiment is indicated in the figure legends. Purification of PBMCs from heparinized blood was performed by density gradient centrifugation (Lymphoprep, Stemcell). Freshly purified PBMCs were washed with RPMI 1640 medium supplemented with 100 U/mL penicillin, and 100 μg/mL streptomycin (P/S, both Sigma-Aldrich) and finally resuspended in medium additionally supplemented with 10% fetal calf serum (FCS, Sigma-Aldrich, complete medium).

### Cell culture and flow cytometry analysis

Flow cytometry experiments were performed in accordance with the “Guidelines for the use of flow cytometry and cell sorting in immunological studies” (Cossarizza et al., 2021). Immunofluorescence surface staining was performed by adding a panel of directly conjugated antibody to freshly prepared PBMCs. Dead cells were excluded from the analysis using a fixable viability dye (Zombie Aqua™ Fixable Viability Kit, Biolegend) or DAPI. After surface staining, cells were fixed and permeabilized using the Cytofix/Cytoperm kit (BD Pharmingen) for TNF, IFNγ, and granzyme B (GrzB) or the eBioscience Foxp3/Transcription Factor Staining Buffer Set (ThermoFisher Scientific) for Tbet and incubated with intracellular Abs. To analyze TNF and IFNγ expression, PBMCs were previously stimulated for 4 h at 37 °C 5% CO_2_ with 30 ng/mL PMA and 500 ng/mL ionomycin in the presence of 10 μg/mL Brefeldin A (BFA, Sigma–Aldrich) or for 16 h with 1 μg/ml anti-CD3 + 10 μg/mL BFA. GrzB expression was assessed in unstimulated cells. Proliferation was measured after stimulation with 1 μg/ml anti-CD3 or 1 μg/ml PHA for 4 days. Samples for flow cytometric analysis were measured using a BD LSR Fortessa X‐20 (BD Biosciences), and analysis was performed with FlowJo software (FlowJo, version 10.8.1). Antibodies used for this study are shown in table 1.

**TABLE 1:** Flow cytometry antibody used for the study.

Antibody	Fluorochrome	Company	Clone
Caspase 3	FITC	BD Biosciences	C92-605.rMAb
CCR7	PECy7	BD Biosciences	3D12
CD158b	PE	BD Biosciences	DX27
CD158e1	PE	BD Biosciences	DX9
CD16	APC	Miltenyi	REA423
CD27	BV421	BD Biosciences	M-T271
CD27	APC	Miltenyi	REA499
CD27	FITC	Miltenyi	REA499
CD28	APC-Cy7	Miltenyi	REA612
CD3	BV650	BD Biosciences	UCHT1
CD45RA	BV510	Miltenyi	REA1047
CD56	APC	Miltenyi	REA196
CD57	PE-CF 594	BD Biosciences	NK-1
CD57	UV	BD Biosciences	NK-1
CD57	BB515	BD Biosciences	NK-1
CD8	PerCP	Biolegend	SK1
CD95	BUV737	BD Biosciences	DX2
CD94	APC	Miltenyi	REA113
CX3CR1	APC	Miltenyi	REA385
GrzB	FITC	Miltenyi	REA226
IFNγ	APC	BD Biosciences	B27
NKp80	APC	Miltenyi	REA845
TNF	PE	BD Biosciences	MAb11

### Cellular senescence assay

Cellular senescence in naïve CD8^+^ T cells was assessed using the Cellular Senescence Detection Kit‐SPiDER‐βGal (Dojindo Molecular Technologies). Bafilomycin A1 was reconstituted in 30 μL dimethyl sulfoxide (DMSO, Sigma Aldrich) and SPiDER‐βGal in 20 μL DMSO (Pangrazzi et al., 2025). PBMCs were incubated in complete medium with a 1:500 dilution of bafilomycin A1 for 1.5 h at 37 °C 5% CO_2_ before the addition of 1:1000 dilution SPiDER‐βGal for another 30 min. PBMCs were washed with PBS and afterwards incubated with surface Abs at 4 °C diluted in PBS.

### Mitochondria assays

Total mitochondrial content was assessed in naïve CD8^+^ T cell subsets within PBMCs using 100 nM MitoTracker Deep Red FM and mitochondrial ROS with 100 nM reduced MitoTracker Red CM-H2-XRos (both ThermoFisher Scientific). Mitochondrial membrane potential was assessed using 0.5 μg/ml JC-1 (ThermoFisher Scientific). Derived PE/FITC mean fluorescence ratio was calculated using Flowjo and was normalised against Mitotracker Deep Red (DR) intensity. PBMCs were incubated in the presence of each dye as well as surface Abs for 20min at 37 °C 5% CO_2_ and afterwards washed with PBS. Negative control samples (i.e., PBMCs stained with JC-1 in the presence of 5 µM FCCP to induce mitochondrial membrane potential depolarization) as well as positive controls (i.e., PBMCs stained with 5 µM oligomycin to induce mitochondrial membrane potential hyperpolarization) were additionally included. After the incubation, cells were washed with PBS and measured immediately at the flow cytometer.

### Measurement of intracellular ROS

To assess intracellular ROS, PBMCs were incubated with the fluorescent dye dihydroethidium (DHE, Sigma-Aldrich) at a concentration of 50 nM in complete RPMI for 20 min at 37 °C 5% CO_2_. Cells were washed in PBS and measured at the flow cytometer.

### Assessment of proliferation

Proliferation was measured after labelling of PBMCs with Cell Proliferation Dye eFluor™ 450 (CPD450, ThermoFisher Scientific) and stimulation with either 1 μg/mL anti-CD3 Ab or 1 μg/mL PHA (BD Biosciences) for 4 days. After incubation, cells were stained with surface Abs and measured by FACS. Results were displayed using the proliferation index (PI, calculated as $\left[\Sigma_{n>1}\left(\frac{P_{n}}{2^{n}}\right)\right]/\;\left[\Sigma_{n>0}\left(\frac{P_{n}}{2^{n}}\right)\right]$ ) and percentage of proliferated cells (i.e., number of proliferating cells/total number of cells) * 100.

### Assessment of apoptosis and DNA damage

Apoptosis and DNA damage were assessed after incubation of PBMCs with 30 μg/mL etoposide (Sigma–Aldrich) for 1 day at 37 °C 5% CO_2_. Apoptotic cells were assessed after intracellular staining with caspase three or alternatively with 400 nM Apotracker green (Biolegend) in the presence of surface Abs and DAPI. Early apoptotic cells were identified as Apotracker^+^ DAPI^−^ and late apoptotic cells as Apotracker^+^ DAPI^+^. DNA damage was quantified after intracellular staining with anti-γH2AX pS139-FITC (REA502, Miltenyi) Ab.

### Statistical analysis

Statistical significance was assessed using nonparametric Friedman test followed by Dunn’s post hoc test, and Spearman correlations, as indicated in the figure legends. A p-value <0.05 was considered significant. Both analyses were performed using the GraphPad 10.1 software. Cluster analysis and related statistics were performed using the JMPpro17 software (SAS Institute Inc., Cary, NC, 2023). Unsupervised clustering was carried out using the ‘hierarchical clustering’ function embedded in JMP environment. The elbow method was used to determine the ideal number of clusters (Thorndike, 1953). Prior to the clustering, the sphericity assumption was confirmed using a Bartlett’s test. The effect size and significant impact of immunological parameters on the clustering model was assessed using a multiple regression model controlling false-discovery rate. Differences in the frequency distributions of cell populations within clusters were evaluated using Fisher’s exact test. Means ± SD are shown in each graph.

## Results

### Differentiation markers, cytokines and effector molecules in N_CCR7_, N_CD28_, N_CD27_ and N_TN_ CD8^+^ T cells

**FIGURE 1:**
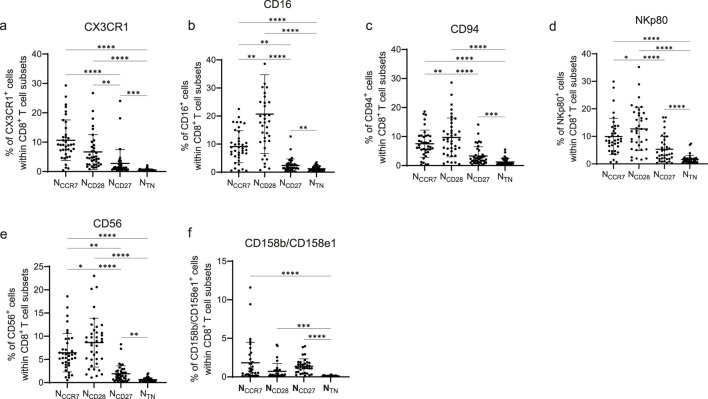
Differentiation markers in N_CCR7_, N_CD28_, N_CD27_ and N_TN_ CD8^+^ T cells. Frequency of **(a)** CX3CR1^+^, **(b)** CD16^+^, **(c)** CD94^+^, **(d)** NKp80^+^, **(e)** CD56^+^, **(f)** CD158b/CD158e1^+^ cells within N_CCR7_, N_CD28_, N_CD27_ and N_TN_ CD8^+^ T cells. n = 39 (CX3CR1, CD16, CD94, NKp80 and CD56) and n = 34 (CD158b/CD158e1) in each subset. Friedman test, Dunn’s post hoc test. *p < 0.05; **p < 0.01; ***p < 0.001, ****p < 0.0001.

To investigate the differentiation state of naïve CD8^+^ T cell subsets, the expression of key T cell differentiation markers was measured in naïve CD8^+^ T cells defined using CD45RA in combination with CCR7 (N_CCR7_), CD28 (N_CD28_), or CD27 (N_CD27_), as well as in phenotypically most “true-naïve”-like CD45RA^+^CCR7^+^CD28^+^CD27^+^CD57^−^ (N_TN_) cells within PBMCs from donors of different age (Figure 1). The gating strategy used to define the four subsets is reported in Supplementary Figure S1. The expression of the chemokine receptor CX3CR1 increases during T cell differentiation (Zwijnenburg et al., 2023). When we measured CX3CR1 levels in the four naïve subsets, it was the highest in the N_CCR7_ subpopulation, and it significantly decreased in N_CD28_, N_CD27_ and N_TN_ cells (Figure 1a). Representative FACS plots showing the levels of CX3CR1 in the four subpopulations is shown in Supplementary Figure S2. The lowest expression was found in the N_TN_ subset, although they were particularly low also in N_CD27_. Natural killer (NK) markers are known to increase with T cell differentiation and show the highest levels in terminally differentiated T cells (Pangrazzi et al., 2020; Koh et al., 2023). The expression of CD16, CD94, NKp80 and CD56 was assessed in the four naïve CD8^+^ T cell subsets and was higher in the N_CD28_ cells compared to the other naïve subsets, slightly lower in N_CCR7_, and further decreased in the N_CD27_ and N_TN_ populations (Figures 1B–E). The NK inhibitory receptor CD158 (CD158b/CD158e1), which belongs to the killer-cell immunoglobulin-like receptor (KIR) family, was absent on N_TN_ cells, while low but still detectable numbers of CD158^+^ cells were found in the other naïve subsets (Figure 1f). Relatively high levels of CX3CR1, CD16, and NKp80 were described in stem-like memory (T_SCM_, CD45RA^+^CD27^+^CCR7^+^CD28^+^CD95^+^) cells (Supplementary Figure Sa-c). Thus, N_CCR7_, N_CD28_, N_CD27_ and N_TN_ show unique expression of differentiation markers.

**FIGURE 2:**
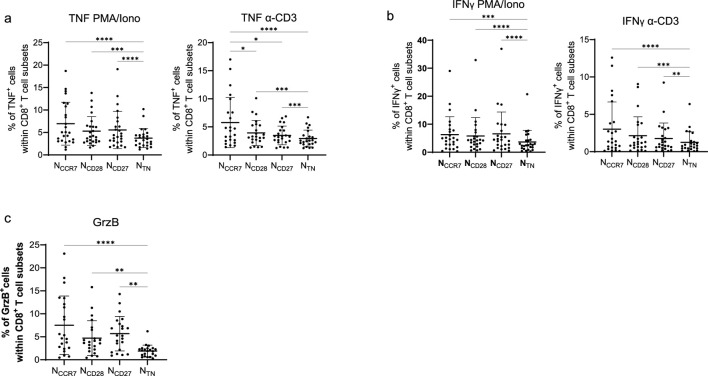
Cytokines and effector molecules in N_CCR7_, N_CD28_, N_CD27_ and N_TN_ CD8^+^ T cells. Frequency of **(a)** TNF^+^, **(b)** IFNγ^+^, and **(c)** granzyme B (GrzB)^+^ cells within N_CCR7_, N_CD28_, N_CD27_ and N_TN_ CD8^+^ T cells. The expression of TNF and IFNγ was assessed after stimulation with PMA, Ionomycin and BFA (PMA/Iono) for 4 h and anti-CD3 and BFA (CD3) for 16 h while GrzB levels were measured in unstimulated cells. n = 25 in each subset. Friedman test, Dunn’s post hoc test. *p < 0.05; **p < 0.01; ***p < 0.001, ****p < 0.0001.

The production of cytokines and effector molecules is associated with T cell differentiation. We therefore investigated whether the expression of TNF, IFNγ, and granzyme B (GrzB) may differ between the N_CCR7_, N_CD28_, N_CD27_ and N_TN_ subsets (Figure 2). In both PMA/Ionomycin and anti-CD3 stimulated cells, TNF expression was highest in N_CCR7_ cells, intermediate in N_CD28_ and N_CD27_ and lowest in the N_TN_ population (Figure 2a). Similar results were observed when IFNγ, and GrzB levels were assessed (Figures 2b,c). Higher expression of TNF, IFNγ, and GrzB was present in the T_SCM_ subpopulation (Supplementary Figures S3d-f). Taken together N_CCR7_, N_CD28_, N_CD27_ and N_TN_ CD8^+^ T cells are different regarding their expression of differentiation markers and T cell cytokines, with N_CD27_ being more like N_TN_ cells.

### Mitochondria, ROS, and senescence in naïve CD8^+^ T cell subsets

**FIGURE 3:**
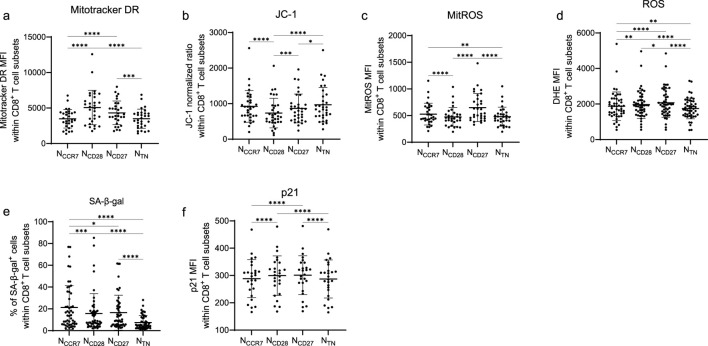
Mitochondria, ROS, and senescence markers in naïve CD8^+^ T cell subsets. **(a)** MitoTracker deep red (DR) mean fluorescence intensity (MFI), **(b)** JC-1 normalized ratio (JC-1 green/red MFI normalized against MitoTracker DR MFI), **(c)** MitoTracker Red CMXRos (MitROS) MFI, **(d)** DHE MFI, **(e)** frequency of SA-β-gal^+^ cells, and **(f)** p21 MFI within N_CCR7_, N_CD28_, N_CD27_ and N_TN_ CD8^+^ T cells. n = 31 (MitoTracker DR, JC-1, MitROS), n = 48 (DHE, SA-β-gal) n = 26 (p21) in each subset. Friedman test, Dunn’s post hoc test. *p < 0.05; **p < 0.01; ***p < 0.001, ****p < 0.0001.

We next measured the expression of parameters related to mitochondrial functionality, oxidative stress, and senescence in N_CCR7_, N_CD28_, N_CD27_ and N_TN_ CD8^+^ T cells (Figure 3). Total mitochondrial content, assessed using mitotracker DR, was highest in N_CD28_ and progressively decreased in N_CCR7_, N_CD27_ and N_TN_ subsets (Figure 3a). Mitochondrial membrane potential, a key indicator of mitochondrial health and activity, was evaluated using JC-1 intensity, normalised on mitochondrial content. While N_CD28_ cells exhibited the lowest JC-1 intensity, it was progressively higher in the N_CCR7_, N_CD27_ and N_TN_ subsets (Figure 3b). The highest JC-1 values were found in N_TN_ cells. Both mitochondrial and intracellular ROS were elevated in N_CD27_ cells, intermediate in N_CCR7_ and N_CD28_, and lowest in the N_TN_ population (Figures 3c,d). Similarly, SA-β-gal activity was higher in the N_CCR7_, decreased in N_CD28_ and N_CD27_ and significantly lower in N_TN_ cells (Figure 3d). Elevated SA-β-gal activity was present in T_SCM_ cells (Supplementary Figure S3g). In parallel, the senescence marker p21 was expressed at comparable levels in the N_CCR7_, N_CD28_, N_CD27_ subpopulations, but it was reduced in the N_TN_ subset (Figure 3e). In summary, the four naïve subsets are unique regarding the functionality of mitochondria and markers of oxidative stress and senescence.

### Apoptosis and proliferation in N_CCR7_, N_CD28_, N_CD27_ and N_TN_ CD8^+^ T cells

**FIGURE 4:**
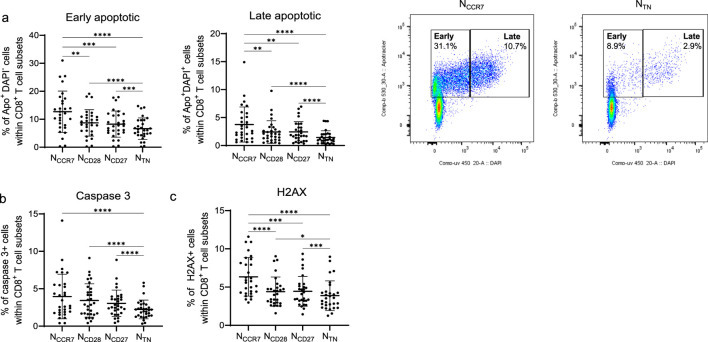
Apoptosis and DNA damage in naïve CD8^+^ T cell subsets. **(a)** Frequency of early apoptotic Apotracker^+^DAPI^−^ (Apo^+^DAPI^−^), and late apoptotic Apotracker^+^DAPI^+^ (Apo^+^DAPI^+^) cells within N_CCR7_, N_CD28_, N_CD27_ and N_TN_ CD8^+^ T cells. Representative FACS plots displaying high (N_CCR7_) and low (N_TN_) apoptosis levels are shown. Frequency of **(b)** caspase 3^+^ and **(c)** γH2AX^+^ cells within naïve CD8^+^ T cell subsets. n = 29 in each subset. Friedman test, Dunn’s post hoc test. *p < 0.05; **p < 0.01; ***p < 0.001, ****p < 0.0001.

**FIGURE 5:**
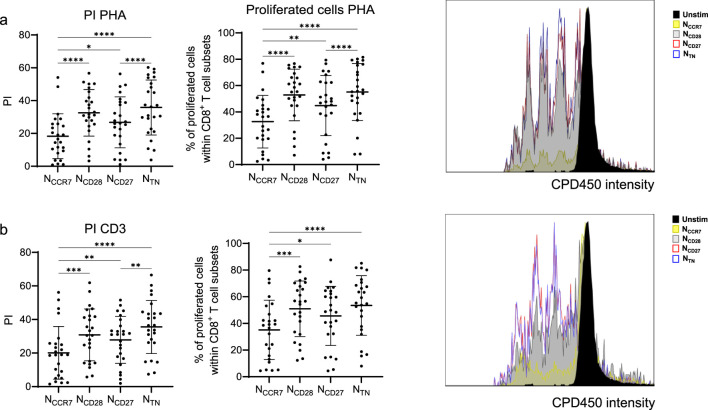
Proliferation potential of N_CCR7_, N_CD28_, N_CD27_ and N_TN_ CD8^+^ T cells. Proliferation potential of CD8^+^ naïve T cell subsets after stimulation with **(a)** PHA and **(b)** anti-CD3 Ab for 4 days. Proliferation was displayed using the proliferation index (PI) and the frequency of proliferated cells. Representative histograms showing CPD450 intensity in the N_CCR7_, N_CD28_, N_CD27_ and N_TN_ subsets in stimulated and unstimulated cells are shown. n = 25 in each subset. Friedman test, Dunn’s post hoc test. *p < 0.05; **p < 0.01; ***p < 0.001, ****p < 0.0001.

We next assessed whether naive CD8^+^ T cells may respond differently to DNA damage (Figure 4). To achieve this aim, PBMCs were stimulated for 1 day with etoposide, and markers of apoptosis were assessed within N_CCR7_, N_CD28_, N_CD27_ and N_TN_ CD8^+^ T cells (Figure 4). We first measured the frequency of early and late apoptotic cells using the combination of Apotracker and DAPI, and therefore Apotracker (Apo)^+^ DAPI^−^ and Apo^+^ DAPI^+^ populations were defined (Figure 4a). The frequency of both early and late apoptotic cells was the highest in N_CCR7_, intermediate in N_CD28_ and N_CD27_ and the lowest in N_TN_ CD8^+^ T cells, indicating that N_TN_ may be more resistant to DNA damage-induced apoptosis. Similarly, the levels of cleaved caspase 3 decreased in N_TN_ cells, although no statistically significant differences were found between N_CCR7_, N_CD28_, and N_CD27_ cells (Figure 4b). γH2AX levels, indicating the presence of DNA double-strand breaks (Podhorecka et al., 2010), were measured in the four subsets, and N_CCR7_ and N_TN_ cells showed the highest and the lowest γH2AX levels respectively. We next measured proliferation of the subpopulations of interest within PBMCs after stimulation with anti-CD3 Ab or PHA for 4 days (Figure 5). Both PI and frequency of proliferated cells were again the lowest in N_CCR7_, intermediate in the N_CD28_ and N_CD27_ subsets, while N_TN_ showed the highest proliferation rate in comparison to the other populations. Results were similar for the stimulation with anti-CD3 and PHA.

Taken together, although the response to DNA damage and proliferative capacities differ across the subsets, the N_CD27_ subset appears most closely related to N_TN_ CD8^+^ T cells.

### Distinct immunological profiles can be defined by differential marker expression in N_CCR7_-N_CD28_ and N_CD27_-N_TN_ cell populations

**FIGURE 6:**
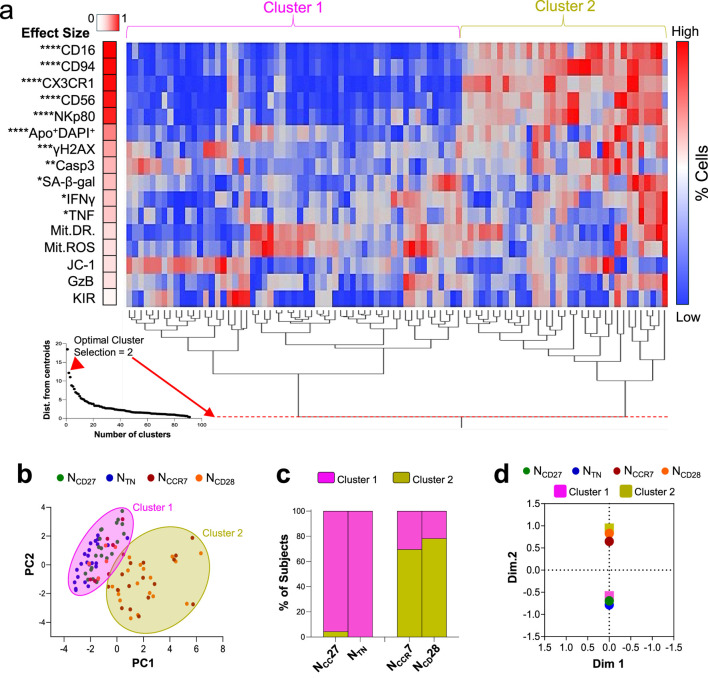
Unsupervised clustering of N_CCR7_, N_CD28_, N_CD27_ and N_TN_ CD8^+^ T cells. **(a)** Hierarchical clustering showing two predominant patterns of expression of immunological parameter. Parameters are arranged vertically according to their discriminative impact on the clustering algorithm. Scatter plot was used to determine the ideal number of clusters to select within the dataset. A model with n = 2 clusters provides the biggest reduction in the distance from centroids. **(b)** Scatter plot allowing the visualization of cluster separation. **(c)** Frequency distribution of cell subpopulations within the theoretical subpopulations identified using unsupervised clustering. Fisher’s exact test p < 0.0001. **(d)** Multiple correspondence analysis confirming the association between N_CC27_ and N_TN_ with cluster one and N_CCR7_ and N_CD28_ with cluster 2.

We then used an unsupervised hierarchical clustering approach to delineate discrete patterns of markers across the entire dataset including the data shown in Figures 1–4 (Figure 6a). In this way, we identified two theoretical clusters of cells (Figures 6a,b). By analysing the relative weight of each marker onto the clustering model, we observed that cluster one showed significantly lower expression of the differentiation markers CX3CR1, CD16, CD56 and NKp80, as well as decreased TNF, IFNγ, SA-ß-gal, and apoptosis. Conversely, cluster two showed an increased level of the same markers. Interestingly, significant differences were additionally driven by apoptosis markers (Figure 6a). Next, cells from the four original subpopulations were identified within the theoretical groups obtained using the clustering algorithm (Figure 6c). Our data show that N_CD27_ and N_TN_ were mostly assigned to cluster 1, whereas N_CCR7_ and N_CD28_ were almost exclusively represented within cluster 2 (Figure 6c). This data was confirmed using the correspondence analysis, showing N_CD27_ and N_TN_ and cluster one in the opposite quadrant as N_CCR7_ and N_CCR8_ and cluster 2 (Figure 6d). Altogether these findings show a remarkable similarity in the phenotype of N_CD27_ and N_TN_, as well as a similar scenario for N_CCR7_ and N_CD28_ subpopulations.

### The effect of age on N_CCR7_, N_CD28_, N_CD27_ and N_TN_ CD8^+^ T cells

**FIGURE 7:**
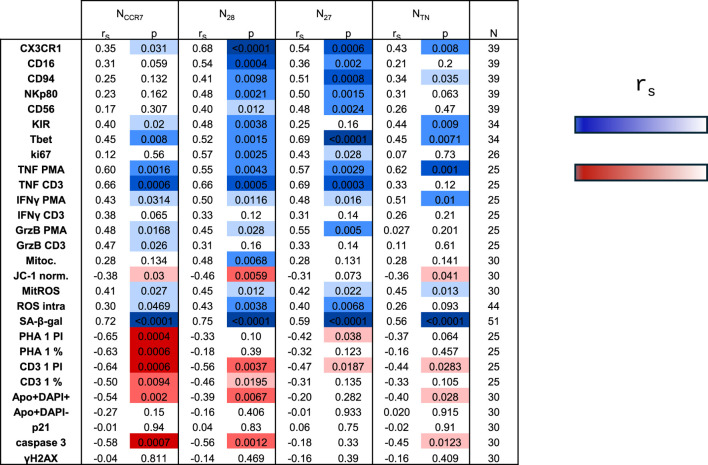
Correlation analysis against age. Correlation of each parameter considered in the study (Figures 1–5) against age. Spearman correlation coefficient (r_S_), p values, and n are shown. For positive correlations, significant p values are displayed using a blue gradient (p = 0.05 light blue, p < 0.0001 dark blue), while for negative correlations p values are shown in red (p = 0.05 light red, p < 0.0001 dark red).

**FIGURE 8:**
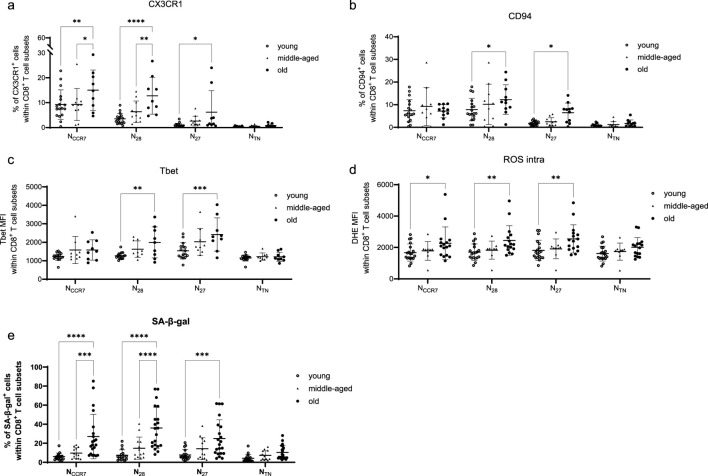
The effect of age on N_CCR7_, N_CD28_, N_CD27_ and N_TN_ CD8^+^ T cells. Expression of **(a)** CX3CR1, **(b)** CD94, mean fluorescence intensity (MFI) of Tbet **(c)** intracellular ROS (=DHE MFI, **(d)**, and **(e)** SA-β-gal^+^ cells in N_CCR7_, N_CD28_, N_CD27_ and N_TN_ CD8^+^ T cells of young (≤35 years), middle-aged (36–69 years), and old (≥70 years) donors. Friedman test, Dunn’s post hoc test. *p < 0.05; **p < 0.01; ***p < 0.001, ****p < 0.0001.

We next investigated whether the expression of all markers measured in the four naive CD8^+^ T cells may change with aging (Figures 7, 8; Supplementary Figure S4). Overall, the expression of differentiation markers and pro-inflammatory/effector molecules increased with age within all subsets, although most significant results were seen for N_CD28_ and N_CD27_ cells (Figure 7). Furthermore, mitochondrial membrane potential was reduced in all subpopulations with age while ROS levels increased. Importantly, in all subsets, highly significant correlations were observed between SA-ß-gal expression and age. Furthermore, the proliferation potential decreased with aging, with the most prominent differences observed for N_CCR7_, N_CD28_, and N_CD27_ cells. Finally, reduced apoptosis was described, although no correlations between γH2AX levels and aging were identified. We next divided the donors into three age groups: young (≤35 years), middle-aged (36–69 years) and old (≥70 years) and investigated the expression of CX3CR1, CD94, Tbet, intracellular ROS levels and SA-ß-gal in N_CCR7_, N_CD28_, N_CD27_ and N_TN_ CD8^+^ T cell subsets (Figure 8). The most significant differences were observed between the young and old groups within the N_CCR7_, N_CD28_ and N_CD27_ subsets, while the middle-aged donors generally resembled the younger group. Although certain trends were present, no significant differences were detected in the N_TN_ subset.

In summary, impaired phenotype and functionality (including the acquisition of an activated phenotype and loss of quiescence) were observed during aging in naive CD8^+^ T cells. Although less pronounced, some age-related changes were also identified in the N_TN_ subset.

## Discussion

CD8^+^ T cells play a key role within the adaptive immune system, enabling the recognition and elimination of intracellular pathogens as well as cancer cells (Appay et al., 2008). After exposure to antigens, naïve CD8^+^ T cells are activated and differentiate into diverse populations of effector/memory CD8^+^ T cells. Complete activation of naïve CD8^+^ T cells requires the expression of costimulatory receptors, including CD28 and CD27, which provide survival and proliferation cues (Dolfi and Katsikis, 2007). Evidence exists that the naïve CD8^+^ T cell pool may be highly heterogeneous regarding phenotype and differentiation status, influenced by factors including thymic function and age (van den Broek et al., 2018). Furthermore, while several studies present in the literature have characterized the phenotype and the functionality of naïve T cells, this subset has been defined in various ways, making proper interpretation of results challenging.

One of the aims of this study was to compare the phenotype of naïve CD8^+^ T cell subsets defined using four different combinations of markers, all expressing the CD45 isoform CD45RA. We considered subpopulations expressing the lymph node homing receptor CCR7 (CD45RA^+^CCR7^+^, N_CCR7_), CD28 (CD45RA^+^CD28^+^, N_CD28_), CD27 (CD45RA^+^CD27^+^, N_CD27_), and cells positive for all markers but lacking the terminally differentiation molecule CD57 (CD45RA^+^CCR7^+^CD28^+^CD27^+^CD57^−^, N_TN_). Although N_CCR,_ N_CD28,_ and N_CD27_ have been partially characterized in other studies (Larbi and Fulop, 2014; van Aalderen et al., 2021), it remains unclear which of these subpopulations exhibit the typical phenotype of *bona fide* naïve CD8^+^ T cells and whether they are phenotypically similar. We therefore analysed the expression of differentiation markers, cytokines, effector molecules, and functional characteristics of the four naïve CD8^+^ T cell subsets within PBMCs from healthy donors of different age. As expected from previous studies (Koch et al., 2008), N_TN_ cells displayed characteristics most closely aligned with naïve cells, as they exhibited very low expression of differentiation markers, pro-inflammatory cytokines and effector molecules, along with excellent mitochondrial fitness, low levels of ROS and senescence markers, reduced DNA damage-induced apoptosis and high proliferation potential after stimulation. Despite this, notable differences among the naïve subsets were observed across all parameters considered. Our analysis of mitochondrial parameters revealed interesting differences among the subsets. N_CD28_ cells showed the highest mitochondrial content but the lowest membrane potential, suggesting unique mechanisms of mitophagy or mitochondrial biogenesis. N_CCR7_, N_CD27_, and N_TN_ cells displayed higher membrane potential, with N_TN_ cells exhibiting the highest values. Importantly, mitochondrial parameters were different between the N_CD27_ and N_TN_ subsets. These findings align with recent studies highlighting the importance of mitochondrial function in T cell differentiation and function (Steinert et al., 2021). Senescence markers (SA-β-gal and p21) showed variable expression across the subsets, with N_TN_ cells consistently showing the lowest levels. This suggests that N_TN_ cells may be less prone to senescence compared to the other subsets. Apoptosis assays suggest that N_TN_ cells may be more resistant to DNA damage-induced apoptosis, which could contribute to their persistence in the naïve T cell pool. Furthermore, N_TN_ cells showed the highest proliferation rate upon stimulation, therefore suggesting that this subset may be most potent to mount immune responses after antigenic contact, therefore being important in responding to infections and vaccinations. To further explore the functional distinctiveness of naïve subsets, we compared the expression of markers associated with activation, senescence, and cytotoxicity between T_SCM_ cells and the other four naïve T cell subsets. Our analysis revealed that the T_SCM_ subset differ markedly from the other subpopulations, frequently exhibiting phenotypic traits characteristic of more differentiated T cells (Tomiyama et al., 2004). These results are consistent with previous studies (Zou et al., 2025; Muroyama and Wherry, 2021) and support the notion that T_SCM_ represent a memory T cell population, clearly distinguishable from naïve T cells both phenotypically and functionally.

**FIGURE 9:**
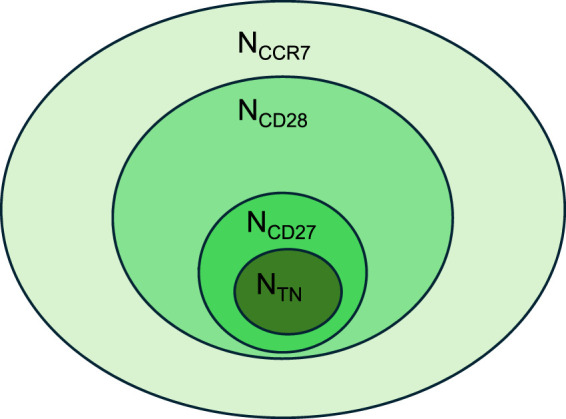
Schematic illustration of the relationship between N_CCR7_, N_CD28_, N_CD27_ and N_TN_ CD8^+^ T cells. Nested diagram showing the conceptual relationship among the four naïve CD8^+^ T cell subsets.

Intriguingly, unsupervised hierarchical clustering identified two distinct clusters: cluster one exclusively including the N_CD27_ and N_TN_ subset, and cluster two predominantly represented by N_CCR7_ and N_CD28_ cells. Importantly, while the levels of differentiation markers, pro-inflammatory cytokines, senescence molecules, and DNA damage-induced apoptosis were particularly low in cluster 1, they were found to be increased within cluster 2. Moreover, the expression of most molecules was highly heterogeneous in the N_CCR7_ and N_CD28_ subsets while being more homogeneous in N_CD27_ and N_TN_ cells. This was particularly evident when assessing the levels of differentiation markers. Thus, these results suggest that N_CCR7_ and N_CD28_ cells likely include other “non-naïve” cell types. Indeed, a small percentage of CD28^−^CD45RA^+^ cells was found within the N_CCR7_ and N_CD27_ subsets while some CD27^−^CD45RA^+^ cells were present within the N_CCR7_ and N_CD28_ subpopulations (Supplementary Figure S5). A schematic illustration showing the conceptual relationship among the four naïve CD8^+^ T cell subsets is shown in Figure 9.

In addition, this heterogeneity may arise from age-related differences in the expression of the parameters of interest. Aging negatively affects the efficacy of immune responses, leading to increased severity of infectious diseases and impaired responses to vaccinations in the elderly (Crooke et al., 2019). Aging significantly impacts naïve T cells, as thymic involution represents one of the earliest and most prominent changes associated with immunosenescence (George and Ritter, 1996). Despite this decline in thymic output, the naïve T cell pool is maintained during aging through homeostatic proliferation mechanisms of existing T cells (Weyand and Goronzy, 2016). A typical aspect intrinsically connected to aging is inflammaging, chronic, sterile, low-grade inflammation developing with aging itself and known to contribute to age-related diseases (Franceschi et al., 2018; Teissier et al., 2022). This persistent pro-inflammatory condition is closely linked to cellular senescence, as senescent cells secrete pro-inflammatory molecules as part of their senescence-associated secretory phenotype (SASP (Coppé et al., 2010)). Importantly, inflammaging alters immune functionality, additionally affecting naïve T cell activation (Shchukina et al., 2023). In particular, a vicious cycle of inflammation may be induced, in which some naïve T cells may be activated by SASP factors and therefore contribute to inflammaging itself with the secretion of pro-inflammatory molecules. Inflammaging directly affects naïve T cell activation, as the pro-inflammatory molecules TNF, IL-6 and IL-1 lead to naïve T cell activation and differentiation (Curtsinger et al., 1999). In addition, altered function of dendritic cells induced by inflammaging itself, as well as impaired formation of the immunological synapse (Tai et al., 2018), further support naïve T cell dysfunction. Consequently, due to inflammaging effects, this subset may show reduced proliferation, increased differentiation towards effector phenotypes, impaired homeostasis and increased susceptibility to exhaustion and senescence (Shchukina et al., 2023). In line with these observations, we documented an overall increase in the expression of differentiation markers, molecules related to inflammation, oxidative stress, and senescence, as well as decreased proliferation and DNA damage-induced apoptosis, in all naïve CD8^+^ T cell subsets during aging. Although stronger correlations were observed in the N_CCR7_ and N_CD28_ subsets, age-related changes were also present in N_CD27_ and N_TN_ cells.

In conclusion, our study reveals a previously unappreciated heterogeneity within the naïve CD8^+^ T cell compartment, with implications for our understanding of T cell differentiation, aging, and immune responses. The N_TN_ phenotype, while still subject to age-related alterations, may provide a more precise definition of naïve T cells compared to the other combinations of markers. In instances where this most optimal definition of naïve CD8^+^ T cells cannot be applied, the combined expression of CD45RA and CD27 may be considered an appropriate alternative. Future studies should assess the phenotype of naïve T cell subsets in pathological conditions. In addition, the functional implications of these subset differences need to be assessed *in vivo*, particularly in the context of aging and immune responses to novel antigens. Additionally, exploring the epigenetic landscape of these subsets could provide insights into the mechanisms underlying their distinct phenotypes and functional characteristics.

### Data limitations and perspectives

Although a small proportion (∼10%) of CX3CR1^+^, CD16^+^, or NKp80^+^ cells was detected within the N_CCR7_ and N_CD28_ subsets, this observation requires further investigation. While our experimental data support the presence of these cells, additional validation is needed to determine whether this reflects a true biological phenomenon or subtle technical limitations related to donor variability or gating parameters. Furthermore, the lack of alternative gating strategies to define naïve T cells (such as CCR7/Fas-based approaches) represents a limitation of the current study.

This study relies primarily on phenotypic analyses using well-established surface markers to define naïve CD8^+^ T cell subsets. Despite comprehensive characterization, these markers may not fully capture the underlying heterogeneity or functional diversity within the naïve compartment. Moreover, potential contamination with partially differentiated or non-naïve cells in some subsets highlights the limitation of surface marker-based definitions. While our hierarchical clustering provided additional resolution, deeper insights could be obtained through high-dimensional approaches such as single-cell RNA sequencing or epigenetic profiling, which were beyond the scope of this study.

## Data Availability

The original contributions presented in the study are included in the article/Supplementary Material, further inquiries can be directed to the corresponding author.
