# Supplementary material for: Phenotypic and functional heterogeneity of naïve CD8+ T cells in human peripheral blood during aging
Source: Front Aging. 2026 Feb 2;7:1765665. doi: 10.3389/fragi.2026.1765665 (PMC12907349; doi:10.3389/fragi.2026.1765665)
Supplement: Supplementary file 1 [file Presentation2.pptx]

## Slide 1
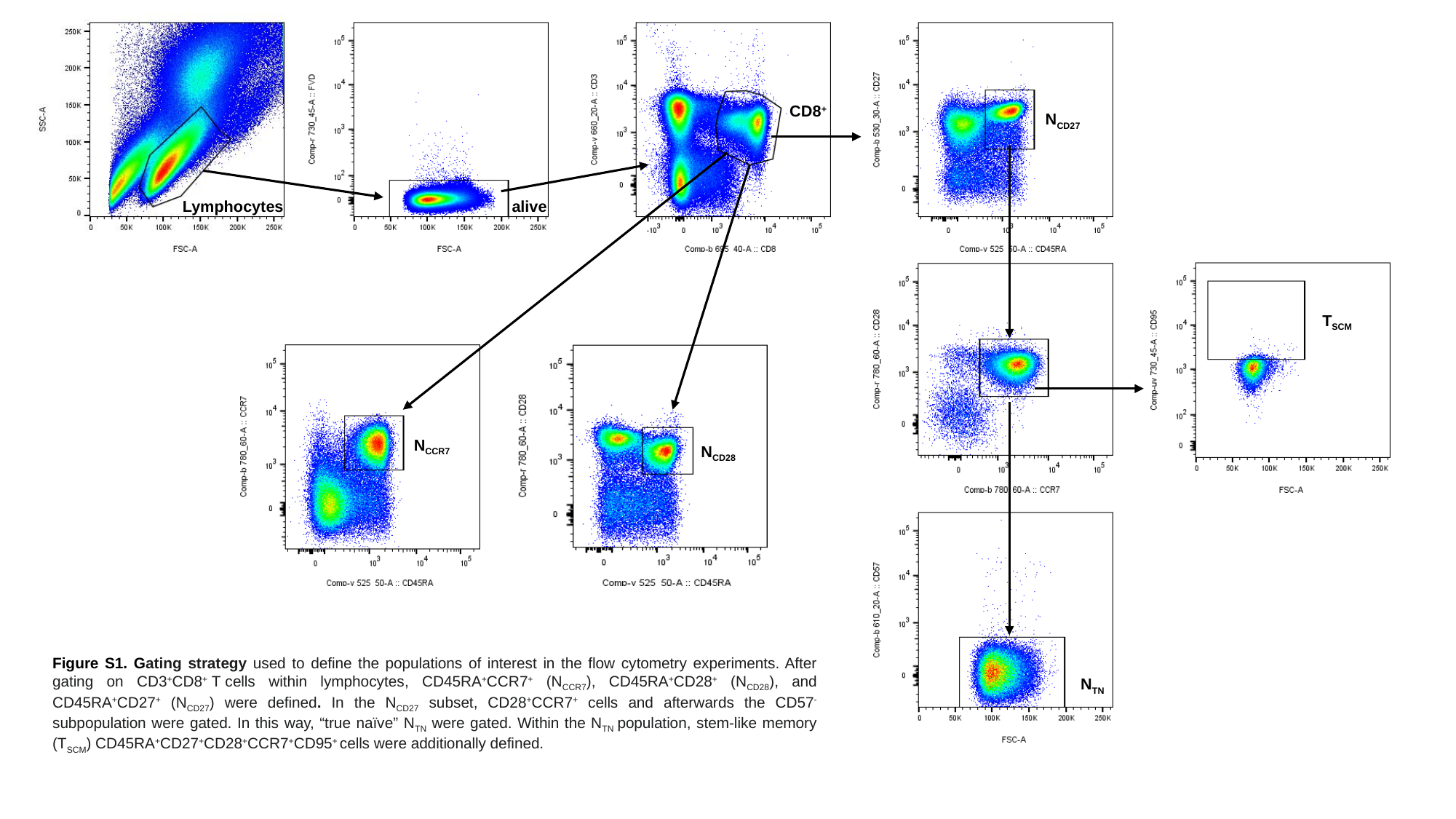

Lymphocytes
alive
CD8+
NCD27
NCCR7
NCD28
NTN
TSCM
Figure S1. Gating strategy used to define the populations of interest in the flow cytometry experiments. After gating on CD3+CD8+ T cells within lymphocytes, CD45RA+CCR7+ (NCCR7), CD45RA+CD28+ (NCD28), and CD45RA+CD27+ (NCD27) were defined. In the NCD27 subset, CD28+CCR7+ cells and afterwards the CD57- subpopulation were gated. In this way, “true naïve” NTN were gated. Within the NTN population, stem-like memory (TSCM) CD45RA+CD27+CD28+CCR7+CD95+ cells were additionally defined.

## Slide 2
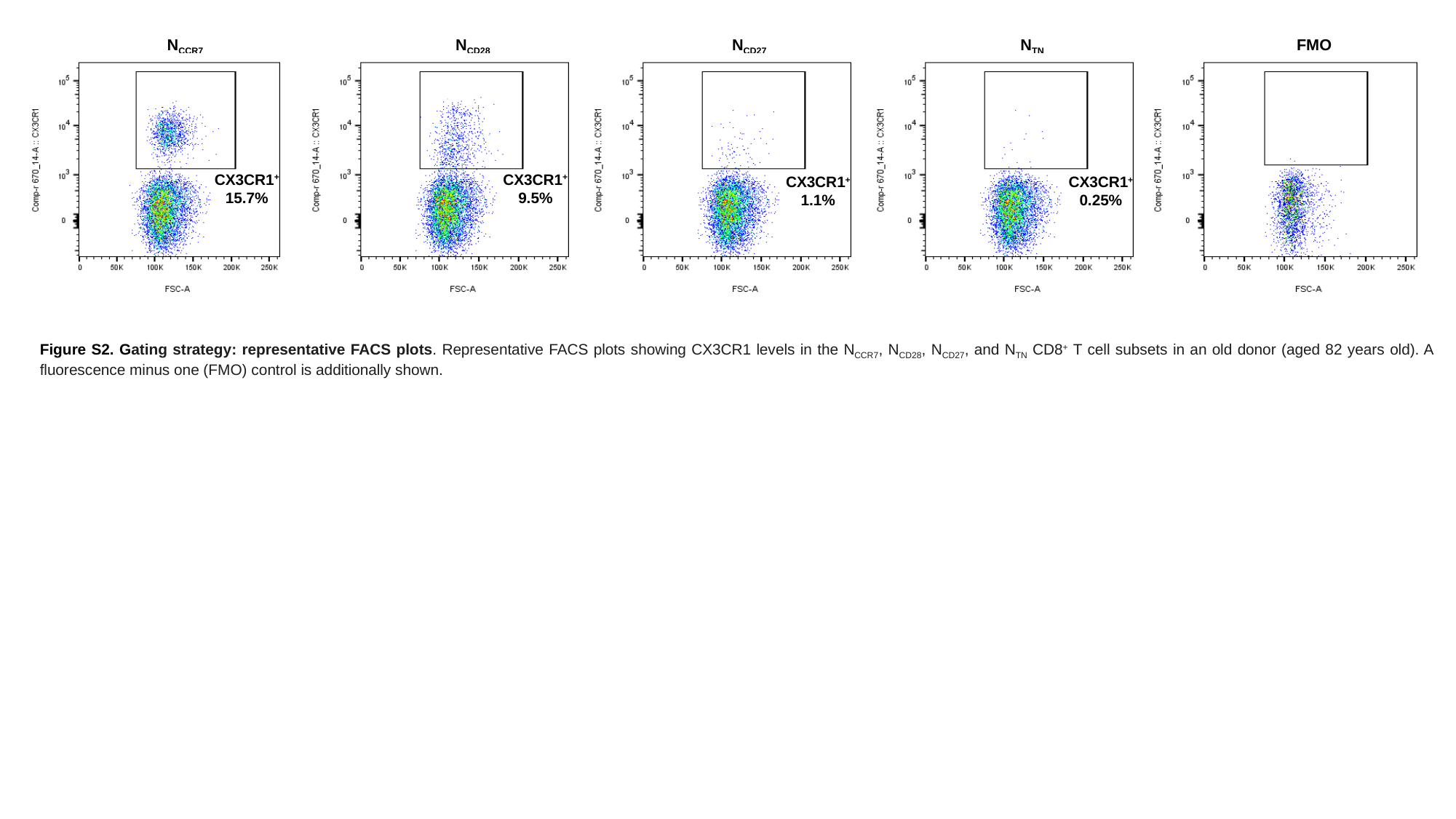

FMO
CX3CR1+
15.7%
CX3CR1+
9.5%
CX3CR1+
1.1%
CX3CR1+
0.25%
NCD27
NTN
NCD28
NCCR7
Figure S2. Gating strategy: representative FACS plots. Representative FACS plots showing CX3CR1 levels in the NCCR7, NCD28, NCD27, and NTN CD8+ T cell subsets in an old donor (aged 82 years old). A fluorescence minus one (FMO) control is additionally shown.

## Slide 3
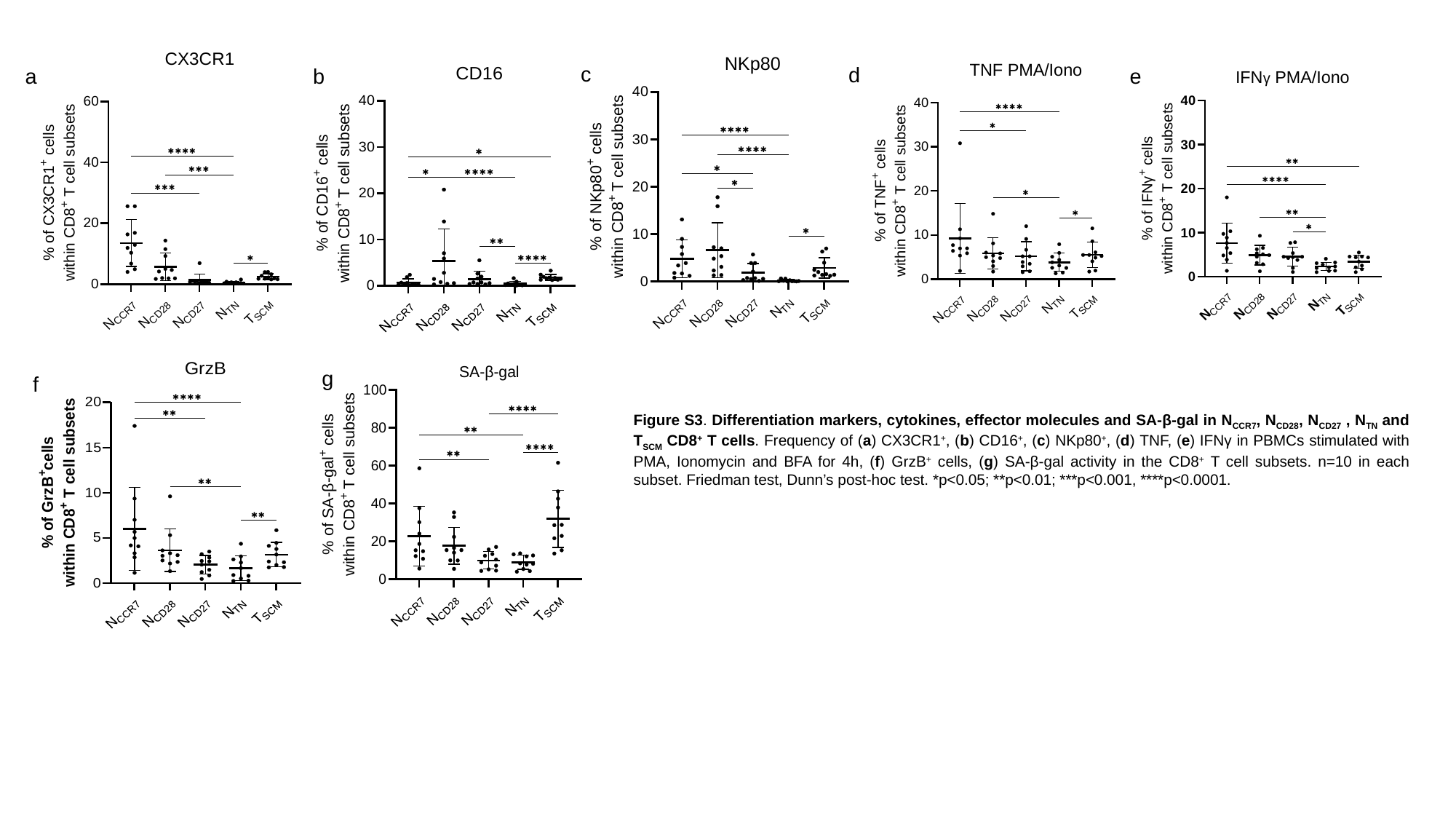

c
d
b
e
a
g
f
Figure S3. Differentiation markers, cytokines, effector molecules and SA-β-gal in NCCR7, NCD28, NCD27 , NTN and TSCM CD8+ T cells. Frequency of (a) CX3CR1+, (b) CD16+, (c) NKp80+, (d) TNF, (e) IFNγ in PBMCs stimulated with PMA, Ionomycin and BFA for 4h, (f) GrzB+ cells, (g) SA-β-gal activity in the CD8+ T cell subsets. n=10 in each subset. Friedman test, Dunn’s post-hoc test. *p<0.05; **p<0.01; ***p<0.001, ****p<0.0001.

## Slide 4
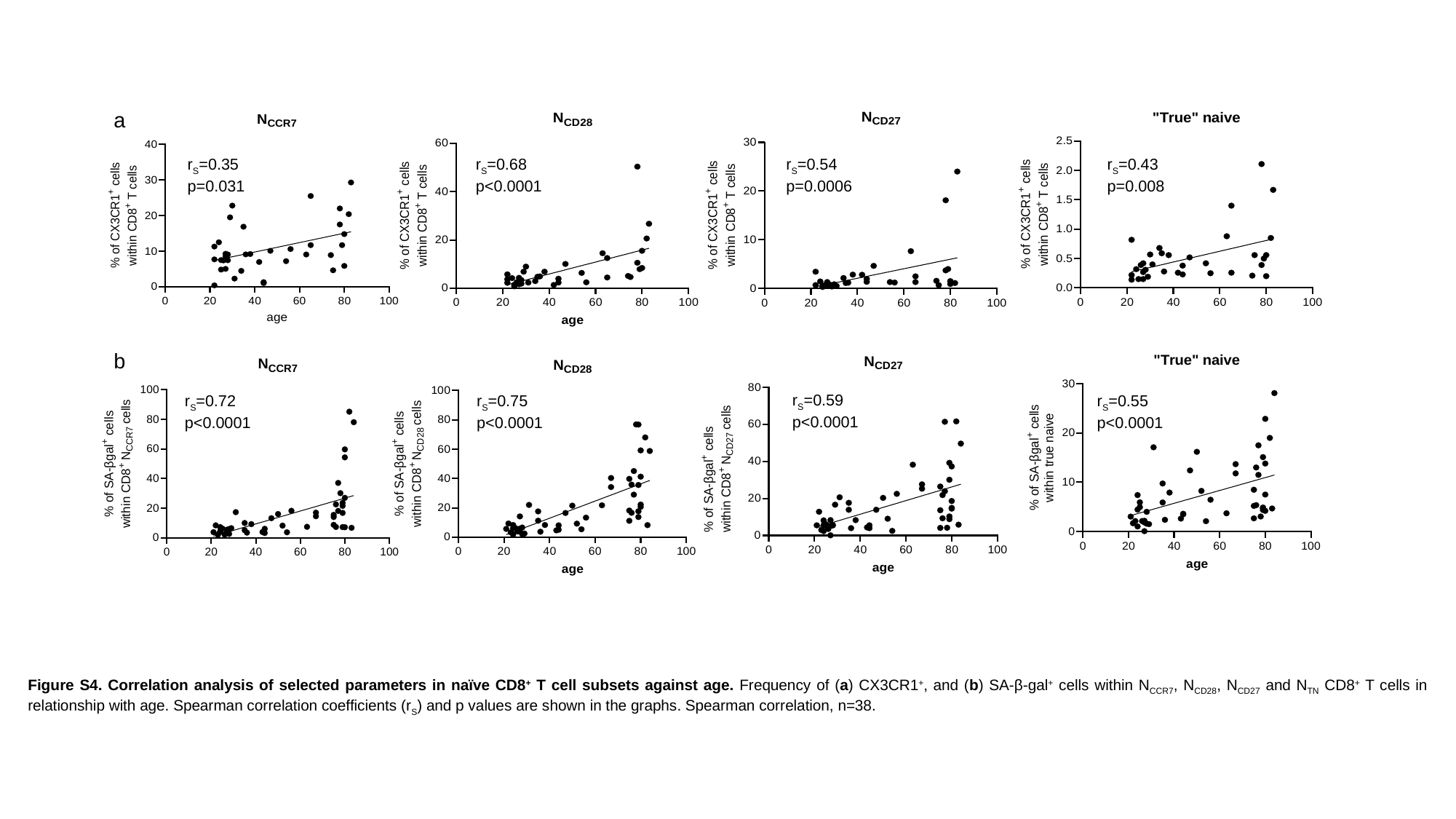

rS=0.35
p=0.031
rS=0.43
p=0.008
rS=0.68
p<0.0001
rS=0.54
p=0.0006
a
rS=0.55
p<0.0001
rS=0.75
p<0.0001
rS=0.59
p<0.0001
rS=0.72
p<0.0001
b
Figure S4. Correlation analysis of selected parameters in naïve CD8+ T cell subsets against age. Frequency of (a) CX3CR1+, and (b) SA-β-gal+ cells within NCCR7, NCD28, NCD27 and NTN CD8+ T cells in relationship with age. Spearman correlation coefficients (rS) and p values are shown in the graphs. Spearman correlation, n=38.

## Slide 5
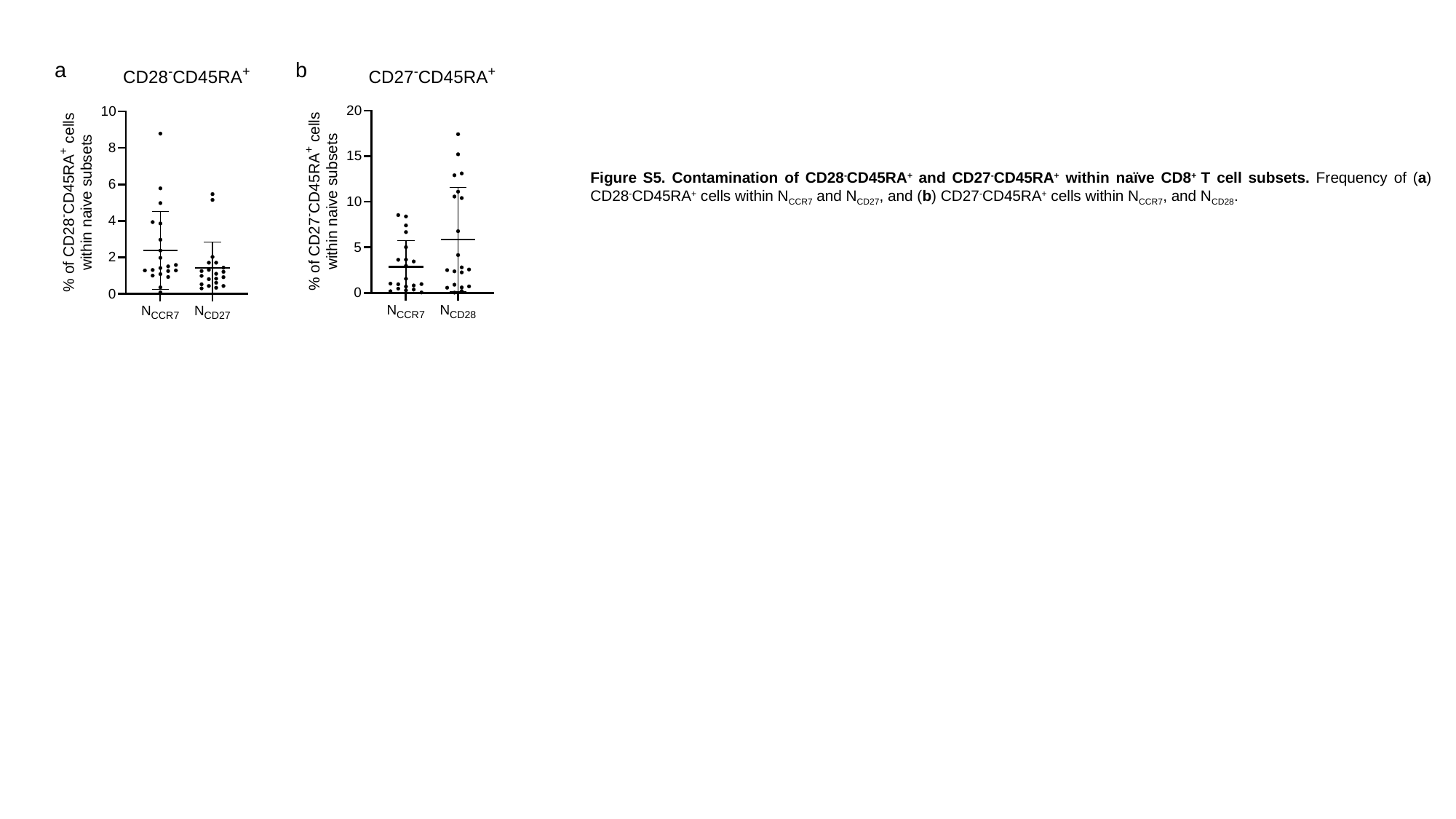

a
b
Figure S5. Contamination of CD28-CD45RA+ and CD27-CD45RA+ within naïve CD8+ T cell subsets. Frequency of (a) CD28-CD45RA+ cells within NCCR7 and NCD27, and (b) CD27-CD45RA+ cells within NCCR7, and NCD28.
